# The growing pains of the 2024/2025 Portugal’s NHS telephone triage system national rollout

**DOI:** 10.3389/fpubh.2025.1694713

**Published:** 2025-10-15

**Authors:** Francisco Goiana-da-Silva, Mário Amorim-Lopes, Fernando Correia, João Pereira, Afonso Ribeiro, Duarte Tude-Graça, Tomás Pessoa-e-Costa, Luís Cabral, Eduardo Costa, Alexandre Morais Nunes, Hutan Ashrafian, Ara Darzi

**Affiliations:** ^1^Centre for Health Policy, Institute of Global Health Innovation, Imperial College London, London, United Kingdom; ^2^NOVA Medical School, Universidade NOVA de Lisboa, Lisbon, Portugal; ^3^Faculdade de Ciências da Saúde, Universidade da Beira Interior, Covilhã, Portugal; ^4^Sword Health, Inc., Draper, UT, United States; ^5^INESC-TEC, Faculty of Engineering, University of Porto, Porto, Portugal; ^6^Neurology Department, ULS Santo António, Porto, Portugal; ^7^AI Research and Development, Sword Health, Inc., Porto, Portugal; ^8^Nova School of Business and Economics, Carcavelos, Portugal; ^9^Faculdade de Medicina, Universidade de Lisboa, Lisbon, Portugal; ^10^Centre for Public Administration and Public Policies, Institute of Social and Political Sciences, University of Lisbon, Lisbon, Portugal; ^11^Dermatology Department, ULS Amadora Sintra, Lisbon, Portugal; ^12^Well Partners Unipessoal LDA, Lisbon, Portugal; ^13^Serviço Regional de Protecção Civil e Bombeiros dos Açores, Angra do Heroísmo, Portugal; ^14^CEGIST—Centre for Management Studies of Instituto Superior Técnico, University of Lisbon, Lisbon, Portugal; ^15^Department of Surgery and Cancer, Faculty of Medicine, Imperial College London, London, United Kingdom

**Keywords:** health system resilience, emergency department, digital health, AI triage, service overload, healthcare optimization

## Abstract

In 2024, the Portuguese NHS introduced “Ligue Antes, Salve Vidas” (“Call Before You Go, Save Lives”), making phone triage through the national SNS 24 line compulsory prior to an Emergency Department (ED) visit in most NHS Local Health Units. While the policy was rightfully concerned with improving timely access to urgent care, it was quickly rolled out without a corresponding investment in infrastructure. The phone triage system’s ability to meet growing call demand was assessed using full population administrative data (January 2024–May 2025), applying a Holt–Winters model to project call volumes. These demand forecasts were compared with a fixed benchmark of installed capacity, allowing for the estimation of unmet demand and waiting times. The authors’ projections show that, without structural reinforcement, the SNS 24 line will face persistent overload, with up to 1 million unanswered calls during the 2025–26 winter season. Unless structural adaptations are implemented, current gaps threaten clinical safety, equity, and public trust. Robust policy options like AI-supported triage, targeted workforce reinforcement, and enhanced transparency are critical to ensure system resilience.

## Introduction

Emergency departments (EDs) are central to modern healthcare systems, providing rapid, multidisciplinary care in acute situations. Yet, in many high-income countries, EDs are increasingly burdened by non-urgent visits or inappropriate for hospital-based care. This misuse leads to diversion of resources from critically ill patients, prolonged waiting times, staff burnout, and compromised continuity of care. In the United Kingdom, for instance, inappropriate ED visits have been estimated to cost the National Health Service nearly £100 million annually (1–3).

Portugal consistently records the highest per capita rates of ED attendance among OECD countries: 2011 with over 70 visits per 100 inhabitants versus an OECD average of 28; 2019 with 79 versus 30; and 2021 with 63 versus 27. Notably, approximately half of all ED episodes are triaged as “green” (standard) or “blue” (non-urgent), indicating cases manageable safely in primary care. These patterns persist despite Portugal’s vast network of primary care services and high ranking in the World Health Organization’s Universal Health Coverage Service Coverage Index, suggesting structural issues in patient navigation and demand management (4–6).

Telephone triage systems have emerged internationally to direct patients to the most appropriate care setting. In countries such as Switzerland, Canada, the Netherlands, and Australia nurse-led triage lines have reduced unnecessary ED visits, often integrated with primary care booking systems and supported by national communication campaigns. Serving as a “digital front door,” these services provide risk stratification, self-care advice, and referral to emergency or specialist services, when needed (7).

Since the 2024 reform, the Portuguese NHS, has been organized into 39 Local Health Units (ULS) (a management model that includes primary and hospital care) and 1 Public-Private Partnership (7).

In 2024, Portugal launched *“Ligue Antes, Salve Vidas”* (*“Call Before You Go, Save Lives”*) requiring patients to contact the national helpline prior to attending hospital emergency departments in selected ULS, which marked a turning point in emergency units’ access (7). Available 24/7, it offers clinical assessment, symptom-based algorithms, referrals and thematic lines, such as “SNS Grávida” for maternal health. Originally a pilot, it quickly expanded to cover 27 ULSs by mid-2025, currently accounting for over 70% of the population (8).

Despite its progressive intent, this policy shift has exposed some system’s design flaws, such as: (a) the lack of substantial adaptation of the triage infrastructure to support exponential increases in call volume, with consequent growth of waiting times prior to emergency calls answers by SNS 24 line operators; (b) the number of calls that are never actually picked up, and (c) triage mistakes with potential associated risks for patients. These issues have been the focus of national media and were even flagged by the National Health Regulator. As this perspective article will argue, the mismatch between policy ambition and operational capacity may negatively impact not only the efficiency but also the safety and credibility of the national digital health infrastructure. Unless this gap is urgently addressed, projections indicate a high likelihood of service overload in the coming winter (9–11).

Operationally, SNS 24 is delivered through a public–private partnership (PPP) between the Portuguese Ministry of Health—via its Shared Services entity (*Serviços Partilhados do Ministério da Saúde*, SPMS)—and a private telecommunications and contact centre operator. The PPP is governed by a multi-year contract that specifies service levels, staffing requirements, maximum call pickup times, and quality assurance mechanisms. Under this arrangement, the private operator provides the technological platform, call centre infrastructure, and the bulk of the human resources (predominantly nurses) required to meet contractual performance indicators. SPMS oversees compliance, conducts audits, and pays the operator based on agreed metrics and volumes (8, 12).

The effectiveness of *Ligue Antes, Salve Vidas* depends on the ability of SNS 24 to manage a rapidly expanding volume of calls without compromising timeliness, quality, or safety. Understanding whether current contractual capacity and scope of services is adequate to sustain this expansion is therefore essential for evaluating the resilience of Portugal’s urgent care access model and for deriving important insights for health systems wishing to replicate this model.

Prior studies on telephone triage and urgent care access have primarily taken a retrospective, descriptive perspective, focusing on past call volumes and service performance indicators [e.g. (13–16)]. While informative, these approaches provide limited guidance for planning under future demand growth. Few works integrate forward-looking demand forecasts with explicit operational capacity benchmarks and transparent uncertainty intervals. This study addresses that gap by combining seasonal demand projections (Holt–Winters exponential smoothing) with a fixed-capacity rule to quantify unmet demand (unanswered calls) and expected waiting times.

## Materials and methods

### Data sources

We assembled four monthly series census-style administrative data covering the entire population of calls to the SNS 24 line, which serves all Portugal’s inhabitants, covering January 2024–May 2025 from the Portuguese Ministry of Health’s *Portal da Transparência* reports titled “Atividade operacional mensal do SNS 24.” These include: (i) total received calls and (ii) answered calls, (iii) average time to answer (seconds), and (iv) unanswered calls. For the population coverage by Local Health Units, we leveraged a dataset from the Portuguese Ministry of Health’s *Portal da Transparência*.

### Statistical analysis

Holt–Winters exponential smoothing was used since it can capture strong seasonality and recent trends typical of respiratory seasons and behavioral shifts. This method is widely used in public-health operations and does not require stationarity. Average wait time per month was defined as a function of seasonality and load on the system, as queueing theory predicts it will rise sharply as the capacity is approached. Diagnostic checks included multicollinearity testing through variance inflation factors, inspection of residual patterns, and heteroskedasticity tests, with robust standard errors in cases where necessary. Full diagnostics are provided in Supplementary Material.

### Projection of received calls

To project future demand, we first modeled the historical series of received calls using an exponential smoothing method (Holt–Winters model). This approach is widely applied in epidemiology and public health planning because it can account for both seasonal fluctuations (e.g., winter peaks in respiratory illness) and recent demand trends (such as the one observed when more ULSs joined). We trained the model on data up to May 2025 and produced forecasts through June 2026, with 80 and 95% prediction intervals to reflect uncertainty. Because by mid-2025 nearly all ULSs had already joined the program, we assumed no further structural increases in demand from new coverage, allowing the model to attribute additional growth to underlying seasonal or behavioral dynamics.

### Installed capacity

To approximate the maximum triage capacity of SNS 24, we adopted a conservative operational definition: the system’s installed capacity was assumed to be equal to the all-time monthly high of answered calls observed in the historical data (January 2024–May 2025). This approach reflects that even though efforts may have been made by the SNS 24 line to increase human resources capacity (17), demand persistently outpaced supply, as reflected by the increased waiting times (18) and concerning volumes of unanswered incoming calls. By anchoring projections to the empirically observed peak, we avoid overstating potential throughput while still allowing for short-term fluctuations in efficiency. This benchmark served as the upper bound of human-staffed performance against which projected call volumes were compared, thereby enabling estimation of unmet demand (i.e., unanswered calls) under different growth scenarios.

### Projection of unanswered calls

Unanswered calls were defined as the portion of projected demand above this capacity. If a month’s forecast stays below 600,000 calls, unanswered calls are zero; if it goes above, the excess is counted as unanswered. The uncertainty bands for unanswered calls were obtained by applying the same rule to the lower and upper ends of the call-volume forecast, then setting negative values to zero. This yields intuitive ranges: zero when demand is comfortably below capacity, and wider bands when demand is close to or above capacity.

### Projection of waiting time

We model average waiting time as a function of load and seasonality, using an OLS specification with non-linear utilization terms. Specifically, we regress waiting time on call volume, utilization and overload terms (including a squared term), and seasonal effects; we exclude 2020–2022 as COVID outliers. The fitted model generates monthly forecasts of waiting time with 80 and 95% prediction intervals that rise sharply as utilization approaches or exceeds capacity, consistent with queueing-theory intuition.

## Results

Building on the publicly available data, according to the previously described methodology, and crossing the resulting trends with the contractual response obligations imputable to the SNS 24 Line actual Operator, we concluded SNS 24 line has been operating above its capacity for at least over 2 years. This statement is based on the fact that, according to the levels of response stated in the above referred binding contract, the target pick up time for any incoming call shall be no longer than 15 s. However, when looking at monthly averages, one may conclude that a considerable amount of incoming calls (at least) did not meet such key service level indicator since the beginning of the actual operation contract. In fact, September 2024 was the month when waiting times were closer to the target, with an average of 23.16 s of waiting times, prior to the national rollout of the “Call before, save lives” project, that made phone triage the new Portuguese NHS emergency Units access paradigm and increased the demand for telephone triage services (19).

Furthermore, our data shows that the historical monthly maximum number of calls answered by the SNS 24 Line has been recorded as of January 2025, reaching a total of approximately 600,000 answered calls. This maximum monthly answered calls value is also used as the maximum projected capacity of the SNS 24 Line when projecting the unanswered calls from May 2025 onwards. In this context, it was confirmed that January 2025 was also the month when approximately 200,000 incoming calls were left unanswered by the SNS 24 Line.

Following the national rollout of the *“Ligue Antes, Salve Vidas”* campaign in 2024, demand has surged dramatically. The project started with a pilot that focused on only a few Portuguese NHS Local Health Units and regions (covering only 8% of the population), in early 2024. However, the rollout of such a project to a total of 27 Portuguese NHS Local Health Units, with a total of 7.9 million potential incoming callers (76% of the population), as well as the Phone Triage Project targeting pregnant women deployed from late 2024 onwards, without due changes and proportionate offer capacity improvements of the SNS 24 Line, had major impacts in the number of unanswered calls by the SNS 24 line. Nevertheless, actually there are still 12 NHS Local Health Units waiting to enroll the telephone triage system, representing a potential additional volume of users of 2.5 million.

**Figure 1:**
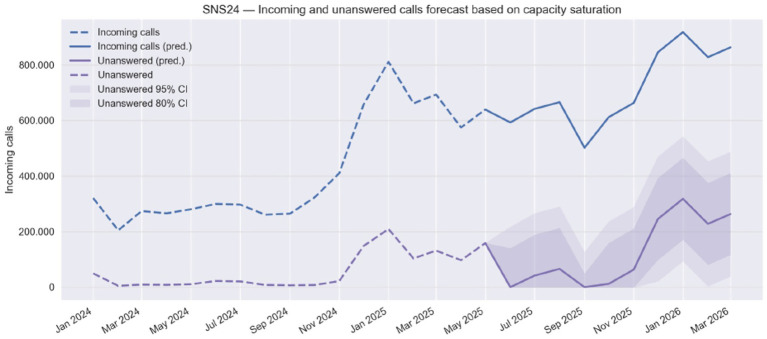
Forecast of incoming and unanswered calls to the SNS 24 line (Jan 2024 – Mar 2026).

Between January and May 2025 alone, attempted calls increased by more than 400,000 per month compared to the same period in 2024 (Figure 1). Our projections for early 2026, anticipate up to 900 thousand monthly call attempts, potentially leaving almost 300,000 calls unattended in a single month. These numbers accrue to over 1 million unanswered phone calls over the entire Winter season and are a conservative estimate, since we are assuming that the compulsory phone triage will not be deployed to the few Portuguese NHS Local Health Units that have not yet joined the program, and that represent a population of approximately 2.5 million patients.

**Figure 2:**
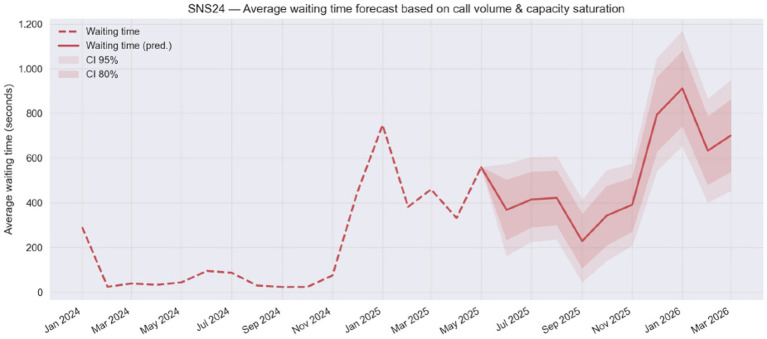
Forecast of average waiting time on the SNS 24 line as a function of call volume and capacity saturation (Jan 2024 – Mar 2026).

On the other hand, when demand spikes, delays increase linearly (Figure 2). We anticipate an impact on answer waiting times peaking in January 2025, with an average of 15.2 min (95% CI: 10.93–19.45). In case the Ministry of Health decides the deployment of the compulsory Phone Triage to all the remaining 12 Local Health Units in Portugal, before the end of the year 2025, the number of unanswered calls as well as waiting times would be considerably aggravated.

## Discussion

The nationwide expansion of the *“Ligue Antes, Salve Vidas”* campaign, while conceptually aligned with international best practices in demand management for urgent care, has not been matched by a proportional reinforcement of the SNS 24 operational capacity. The current public–private contract between the Ministry of Health (through SPMS) and a private communications provider defines explicit service-level obligations, including maximum average call pickup times, minimum answer rates, and continuous availability for specific priority lines. However, publicly available performance data show persistent non-compliance with these benchmarks, particularly regarding the 15-s maximum pickup target for incoming calls, which has never been met since the start of the current contractual cycle according to monthly averages (12).

The situation is aggravated by the recent launch of additional specialized services under the SNS 24 umbrella, such as the SNS Grávida line. Testimonies from SNS 24 working nurses reported by national media in August 2025 indicate that the promised staffing by maternal health specialists has not materialized, with generalist nurses receiving only minimal self-directed training and reporting difficulty in safely triaging obstetric calls (10). This undermines both clinical safety and contractual obligations for adequate staffing, particularly for services publicly promoted as specialist-led.

From a systems perspective, the mismatch between policy ambition and operational reality carries three major risks:

1. *Performance degradation and reputational erosion*—Prolonged wait times and unanswered calls jeopardize the public’s willingness to comply with the “Call First” mandate, risking policy reversal and behavioral relapse toward direct ED attendance (20–23).
2. *Spillover to emergency services (INEM)*—When SNS 24 queues saturate, patients increasingly bypass the system and call the 112 emergency line, diverting capacity from true emergencies. Early 2025 data already show a measurable increase in INEM call volumes. In fact, in the first half of 2025, INEM calls increased by 8.6 and 20% compared to the first half of 2024, and to the first half of the years between 2012 and 2024, respectively. Such an increase may be plausibly linked to the increase in SNS 24 calls and waiting times (24). However, it is not possible to exclude that other institutional and behavioral determinants may also have played a role in such outcome.
3. *Equity and safety threats*—Extended waits or failed connections disproportionately affect vulnerable groups with fewer digital alternatives or access to private healthcare facilities, potentially delaying recognition of life-threatening conditions (25, 26). Furthermore, the fact that Emergency Units access qualification through Telephone Triage is actually only implemented in 27 from the 39 NHS Local Health Units, with pending additional rollouts hypothetically due to lack of response capacity by SNS 24 Line, creates important asymmetries between different regions of the country, undermining the possibility of flawless and transversal nationwide communication and literacy campaigns for this purpose.

The legal and procurement framework further constrains rapid capacity expansion. Under Portuguese and EU public procurement law, any substantial change in contract scope whether by volume, budget, or service specification, may trigger mandatory re-tendering, a process typically spanning several months. This creates a structural barrier to scaling the existing human-based triage model in time to meet projected and usual winter peaks.

To safeguard the policy’s objectives and prevent significant operational overload during the 2025–2026 winter season, urgent action is required. This could be achieved in three main ways:

1. *Automated triage through AI*—AI-driven triage systems have the potential to support healthcare professionals in high-pressure environments, namely through absorption of low-acuity demand and allow human operators to focus on complex and high-risk cases. AI-driven symptom checkers and conversational agents, already validated in countries such as the UK, Finland, and Australia, can manage high concurrency, filter low-complexity cases, and escalate to human operators when needed. Such integration would preserve the human element for high-risk and complex presentations, while substantially increasing throughput at lower marginal cost and within current legal frameworks. As healthcare demands continue to grow, these systems represent a vital innovation for advancing emergency care and addressing longstanding challenges in triage (27–29).
2. *Targeted workforce reinforcement*—Expand staffing with fast-track, competency-based training, particularly for new specialist lines such as SNS Grávida [(30, 33, 34)].
3. *AI Continuous Auditing and Reporting Tools*—Progressively replace traditional auditing processes based on sample calls and acts analysis by AI auditing systems, able to supervise and check the compliance of all acts/calls, 24/7 in real time, therefore improving reporting and prevention of harm incidents. In an operation characterized by millions of calls, every day, patient safety incidents occur that go unrecorded. Without comprehensive incident reporting, it is very difficult to learn from patient safety incidents and improve prevention and response. AI Auditing and reporting tools can automatically populate incident report forms, categorize incidents according to a set taxonomy and identify trends across reports. Not only is this a significant time-saver for an already strained workforce, but as the model becomes even more advanced, AI can help identify and analyze trends, enabling better-informed decision-making and creating a safer environment for all (27, 29, 31).

In parallel with these initiatives, additional efforts should be undertaken regarding transparency, namely through:

4. *Improve reporting transparency***—**by reporting detailed, disaggregated KPIs rather than aggregated averages or absolute volumes. For example, instead of only showing monthly average call wait times, report in addition minimum, maximum, median values, and full distribution curves. This would not only increase transparency but also provides an opportunity to gain deeper operational insights, identify patterns, and optimize resource allocation (1, 3, 7).
5. *Publication of audit results***—**Regular public release of findings from independent audits of the operator’s performance, including compliance with contractual obligations, training standards, and staffing levels (32).

In addition, consideration should be provided to *accountability through enforcement*, namely through the application of contractual penalties for persistent non-compliance, particularly for failure to meet pickup time targets, quality standards, or specialist staffing requirements (28).

In the medium term, legal reforms should be considered, to modernize future procurement frameworks to allow agile scaling of digital health services, incentivizing AI supported processes for more efficient, safer and expandable services (3, 28).

Nevertheless, this analysis is subject to important limitations with regards to the data used (including reliance on aggregated monthly administrative data, absence of patient-level clinical outcomes and of data on informal demand bypassing the system), the modelling assumptions (namely the static maximum capacity), and finally, the projections’ sensitivity to external factors that impact SNS24 activity (unforeseen and variable peaks in seasonal epidemics, public policy changes, are both potentially relevant examples). Our analyses are descriptive and predictive rather than causal. Associations between load and waiting time are consistent with queueing theory but should not be interpreted as causal effects. Alternative explanations for the observed patterns include changes in call-handling protocols, staffing mix, and public communication.

The use of aggregated administrative data restricts granularity, preventing analysis of patient heterogeneity, call categories, or clinical outcomes, with the latter being directly and negatively affected by lower call answer rates and higher waiting times. Administrative data may also contain measurement error (e.g., time-stamping or categorization). Although routine quality checks are in place, minor discrepancies cannot be ruled out. Informal demand bypassing the system (e.g., patients going directly to emergency services) is also not captured, leading to possible underestimation of true demand.

Installed capacity was defined as the historical maximum of answered calls represented as a straight horizontal red line on Figure 1, which may reflect temporary overextension rather than sustainable throughput. Similarly, proportional scaling of demand for the remaining ULS populations may not hold if regional demographic, socioeconomic, or digital literacy profiles differ. External validity is bounded by the organization of SNS 24 while the forecasting method is portable, parameters are system-specific.

These constraints do not undermine the main conclusions but suggest caution in extrapolating projections to other contexts.

## Conclusion

In 2024, the Portuguese NHS introduced “Ligue Antes, Salve Vidas” (“Call Before You Go, Save Lives”), making phone triage on the national SNS 24 line compulsory prior to an emergency department visit in most NHS Local Health Units. After encouraging outcomes of the pilot project of “Ligue Antes, Salve Vidas” project, it was quickly rolled out without corresponding investment in infrastructure. This opinion piece analyzes full-population administrative data to illustrate how growing demand has continually exceeded the SNS 24 line’s capacity, leading to hours of waiting, unheard calls, and successive breakdowns in contractual service level targets. The authors believe these issues are the outcome of subpar rollout planning and the SNS 24 line operator failing to ramp up resources to meet national coverage, particularly during peak periods.

Besides documenting these operational challenges, the study provides a theoretical contribution by presenting an additional and transparent approach to transform routine call data into capacity-sensitive estimates of unmet demand and waiting times. The use of Holt–Winters forecasting combined with a fixed-capacity rule and fan-chart uncertainty bands demonstrates how descriptive administrative series can be repurposed as an easy-to-use planning tool to enhance health system resilience.

Short-term implications include reinforcing staffing levels, improving surge-readiness for winter peaks, and ensuring adequate coverage of specialist lines such as maternal health. Longer-term implications concern structural reforms in capacity planning, the integration of AI-enabled triage and auditing systems, and greater transparency in performance reporting.

Unless addressed, the disparity threatens the clinical safety, public trust, and equity goals of the initiative. This winter, the authors’ projections demonstrate a high probability of system overload. There is a call for urgent structural responses both from the Portuguese Ministry of Health and by the SNS 24 Line Private operator to facilitate sustainable system resilience by adopting AI-powered triage and audit tools, strengthening the workforce specifically for specialist lines such as maternal health and more openness with disaggregated reports on performance as well as real-time release of audit results. In order not to lose the cultural change benefits the “Ligue Antes, Salve Vidas” project drove, decision makers shall protect its building principles and drive due structural adaptations for it to thrive.

## Data Availability

The original contributions presented in the study are included in the article/supplementary material, further inquiries can be directed to the corresponding authors.
